# Innovation in wild Barbary macaques (*Macaca sylvanus*)

**DOI:** 10.1038/s41598-020-61558-2

**Published:** 2020-03-12

**Authors:** Federica Amici, Alvaro L. Caicoya, Bonaventura Majolo, Anja Widdig

**Affiliations:** 1 Behavioral Ecology Research Group, Institute of Biology, Faculty of Life Science, University of Leipzig, Leipzig, Germany; 2 Research Group Primate Behavioural Ecology, Department of Human Behavior, Ecology and Culture, Max-Planck Institute for Evolutionary Anthropology, Leipzig, Germany; 3 Department of Clinical Psychology and Psychobiology, Faculty of Psychology, University of Barcelona, Barcelona, Spain; 4 School of Psychology, University of Lincoln, Lincoln, United Kingdom

**Keywords:** Evolution, Psychology, Zoology

## Abstract

Innovation is the ability to solve novel problems or find novel solutions to familiar problems, and it is known to affect fitness in both human and non-human animals. In primates, innovation has been mostly studied in captivity, although differences in living conditions may affect individuals’ ability to innovate. Here, we tested innovation in a wild group of Barbary macaques (*Macaca sylvanus*). In four different conditions, we presented the group with several identical foraging boxes containing food. To understand which individual characteristics and behavioural strategies best predicted innovation rate, we measured the identity of the individuals manipulating the boxes and retrieving the food, and their behaviour during the task. Our results showed that success in the novel task was mainly affected by the experimental contingencies and the behavioural strategies used during the task. Individuals were more successful in the 1-step conditions, if they participated in more trials, showed little latency to approach the boxes and mainly manipulated functional parts of the box. In contrast, we found no effect of inhibition, social facilitation and individual characteristics like sex, age, rank, centrality, neophobia and reaction to humans, on the individuals’ ability to innovate.

## Introduction

Innovation has been defined as the solution to a novel problem, or the novel solution to a familiar problem^1,2^. In humans, the ability to innovate and socially transmit innovations has been crucial through evolution, allowing us to reach a unique level of behavioural and cultural complexity^3,4^. However, innovation also provides a variety of benefits to other animals, allowing them to develop novel responses against predators, exploit novel resources and invade new niches^1,5–15^. By allowing individuals to better cope with novel socio-ecological challenges, innovation is thought to provide direct fitness benefits, especially in dynamic environments^1,2,9,10,12,13,16,17^

The ability to innovate is therefore widespread across taxa. Several species of birds, fish, carnivores and rodents, just to name a few, show evidence of innovation^2,18,19^ for reviews. Studies in captivity have also shown that some primate species are excellent problem-solvers^20–27^. Great apes, for instance, can use several new solutions to extract food from a container, inhibiting old strategies when they become inefficient^24^. When presented with a novel foraging task, great apes could successfully move food through a maze, planning up to two steps ahead to avoid food falling into traps^28^. Even lemurs (*Varecia* spp.) and callitrichid monkeys (*Leontopithecus* spp., *Saguinus* spp., *Callithrix* spp.), who are phylogenetically more distant from humans and have relatively small brain sizes^29^, can solve novel foraging tasks by retrieving food from different containers^21^.

Apart from inter-specific differences, there are also several intra-specific differences in terms of innovation. Traditionally, these differences have been explained by the Innovation by Necessity and the Free Time hypotheses^2,30–32^. The Innovation by Necessity hypothesis (also called Bad Competitor hypotheses^18^), in particular, predicts that innovation is especially frequent in those individuals who would otherwise have no access to food, and thus need to take the risk of performing potentially dangerous novel behaviours as a last resort^2,31,32^. According to this hypothesis, better innovators would be individuals in bad physical condition, lower-ranking or younger, and in species with sexual dimorphism, individuals of the smaller sex (e.g. birds^13^ and primates^33,34^). In contrast, the Free Time hypothesis (also called the Excess of Energy hypothesis^18^) predicts that innovation is more frequent when individuals are fit and healthy, and have more time and energy to devote to innovation^30^. Therefore, innovation should be more frequent in higher-ranking animals, younger individuals and animals of the larger sex (e.g. birds^35–37^, carnivores^38,39^ and primates^40,41^).

Moreover, sociality may increase fitness and be positively linked to innovation. Strong and enduring social relationships, for instance, play a crucial role in animal life, and predict individual fitness in both human^42,43^ and non-human primates^44–49^. It is therefore possible that individuals having many social interactions, or those who are “social hubs” in the group^50^, may be more fit and prone to take the risks linked to innovation, as they can count on extended social support (in line with the Free Time hypothesis). In theory, it is also possible that more innovative individuals increase their prestige or status through the implementation of novel solutions, so that they are preferred over other social partners^51,52^. In both cases, high sociality could be predictive of individual innovation rate. In line with the Innovation by Necessity hypothesis, in contrast, low sociality would imply lower individual fitness, and thus higher innovation rate.

A recent meta-analysis on innovation in mammals and birds^18^, showed that individuals of the larger sex innovate more than those of the smaller sex, supporting the Free Time hypothesis; none of the other factors proposed by the Innovation by Necessity and the Free Time hypotheses predicted innovation. Therefore, it is likely that factors other than sex, age, rank or physical condition may more reliably predict the distribution of innovation. For example, individuals strongly differ in their tendency to avoid or approach novel objects (i.e. neophobia or neophilia, respectively), which may clearly affect their participation in novel tasks and their ability to solve novel problems^20,39,53–60^. Response to novelty usually correlates across different contexts^60–63^, and researchers rely on different methods to assess it (e.g., reaction to novel objects, food or environments).

Similarly, some individuals are more persistent than others, spending more time interacting with and attempting to solve novel tasks^19^. Individuals also differ in the variety of exploratory behaviours shown when interacting with the novel task, and in the number of objects or parts of the objects manipulated^18^. These differences in persistence and exploration have been shown to affect innovation^2,19,20,38,39,53,56,59^. In orangutans (*Pongo pygmaeus* and *P. abelii*), for instance, the ability to solve novel problems is especially high for individuals who are highly explorative, and show a positive response to novel objects and humans^64^.

To date, the majority of experimental studies on innovation has been conducted in captivity, where controlled conditions are often easier to implement. In the wild, innovation has been mainly studied by conducting observations to find instances of novel behaviours^65–68^. However, innovations in the wild are more sporadic and less predictable, and very infrequent behaviours might be wrongly classified as innovations simply because they had never been observed before^1,2,69,70^. An alternative approach is thus to study wild animals with novel experimental tasks. Some studies have successfully implemented this experimental approach, testing innovative behaviour in different species of wild birds^36,60,71–74^ and carnivores^38,75^. To our knowledge, however, such an experimental approach to the study of innovation has only been used twice in primates. Huebner and Fichtel^76^ tested wild red-fronted lemurs (*Eulemur rufifrons*) on a foraging box that could be opened in three different ways. As soon as one solution had been mastered, it was blocked and individuals had to invent another one, inhibiting the previously learned strategy. In this study, most individuals interacted with the boxes, and the best innovators were adults and more explorative individuals^76^. In another study, Laidre^40^ presented a group of baboons (*Papio anubis*) with three different novel tasks, in which individuals could access out-of-reach food by either (i) pulling a string attached to bananas, (ii) dipping with sticks in a metal tube, or (iii) using a stick to push food out of a metal conduit. Baboons could only solve the first task, and failed with the other two even when the sticks had been already positioned in the correct position to facilitate solution of the task.

These results contrast with those on captive primates, and suggest that captive individuals may be more innovative than their wild counterparts (in birds^36,60,77^ and hyenas^39^). In captivity, animals experience atypical living conditions, which may affect the development of their cognitive skills^1,60,77–79^. Continuous exposure to the human cultural milieu and to novel objects, in particular, may provide captive individuals with more chances to understand the physical properties of objects and their potential function as tools^40,80,81^, or even simply reduce their neophobia toward humans and experimental set-ups^56,64,82–84^. Therefore, collecting more information on wild primates, by testing them in their social groups under natural conditions, appears a necessary step to understand how the ability to innovate really varies across conspecific individuals and to assess whether the same evolutionary pressures are at play across different taxa.

In this study, we aimed to test innovation in a wild group of Barbary macaques (*Macaca sylvanus*). In four different conditions, we presented the study animals with several identical foraging boxes. In each condition, individuals had only one way to open the box. While non-functional parts of the boxes were completely transparent, functional parts were coloured green across all conditions. We then measured the identity of the individuals participating and solving the novel task, and their behaviour during the task (i.e. latency to interact with the box, time spent manipulating non-functional and functional parts of the boxes, and number of attempts to reach the food through the transparent plexiglas). We further measured individual levels of neophobia while feeding on novel food, and conducted behavioural observations to assess individual rank, position in the social network and reaction to humans. We then analysed whether innovation rate was affected by the individual behaviour during the task (as defined above), and whether individual characteristics (i.e. sex, age, rank, neophobia, position in the social network and reaction to humans) affected innovation rate and behaviour during the task. Detailed predictions are summarized in Table 1.

**Table 1:** Predictions about the individual characteristics and behaviours which might predict success in the innovation task by the wild Barbary macaques we tested (predictions confirmed by our results are in bold).

Hypotheses	Sex	Age	Rank	Centrality	Neophobia	Reaction to humans	Latency	Exploration	Functional manipulations	Inhibition	Social facilitation
Free Time	Males	Young	High	High							
Innovation by Necessity	Females	Young	Low	Low							
Personality					Low	Positive					
Behaviour							**Low**	High	**High**	High	Presence of others

## Methods

### Subjects

We studied 19 Barbary macaques (*Macaca sylvanus*) living in a free-ranging group in Gibraltar. The group included males and females of different age classes and rank (see Table 2 for more details). The monkeys lived in an area characterized by steep cliffs and sparse vegetation, in a military zone where tourists and inhabitants have no access. The group foraged in the area, but was also partially provisioned with small quantities of fruit and vegetables every morning, which covered a limited part of their diets. In this way, macaques still had to forage, but they limited their incursions in the village to search for food.

**Table 2:** List of the wild Barbary macaques located in Gibraltar and participating in the study.

Subject	Sex	Age class	Elo-rank	RH	Neophobia	Centrality	Strength	Trials
Batmana	female	adult	0.53	2	0.50	0.47	24	8
**Chicho**	male	juvenile	0.69	2	0.48	0.90	41	97
Colega	male	adult	0.22	2	0.67	0.65	30	9
**Gruñon**	male	adult	0.67	3	0.48	0.38	18	27
**Jefa**	female	adult	0.60	3	0.50	0.72	34	18
Legolashijo	male	adult	0.51	3	1.00	0.20	7	0
Legolaspadre	male	adult	0.77	2	0.47	0.93	41	10
Mamabebeenano	female	adult	0.00	4	0.45	0.66	32	3
Mamanoel	female	adult	0.76	2	0.60	0.54	26	37
Mancha	female	adult	0.58	3	0.50	0.15	7	0
**Mephisto**	male	adult	0.88	3	0.54	0.59	27	17
**Nerd**	female	adult	0.25	4	0.50	0.09	4	23
**Noruega**	female	adult	0.48	3	0.37	0.80	40	36
Orejacortada	female	adult	0.37	1	0.64	0.75	35	13
Paul	male	adult	1.00	3	0.48	1.00	49	21
Pedro	male	juvenile	0.39	2	0.50	0.71	33	13
Pendiente	male	adult	0.11	4	1.00	0.31	15	0
Tetas	female	adult	0.06	4	0.40	0.33	16	19
Uniteta	female	adult	0.51	3	0.61	0.52	23	45

We classified as adults all females estimated to be above 5 and all males above 6, while juveniles where individuals between 1 and 3 years of age. One corresponds to a high rank, a positive reaction to humans (RH), and high neophobia. More central individuals and individuals with higher strength have higher values. Trials indicated the number of trials in which individuals participated. In bold, individuals who retrieved food in at least one trial.

### Materials and procedures

Between October 2017 and March 2018, we conducted behavioural observations on all adults and subadults in the group, whilst no experiment was administered. Firstly, we used the Elo method to determine the dominance hierarchy (EloRating package, version 0.43), based on all dyadic agonistic interactions with a clear winner-loser outcome, recorded via all occurrence sampling^85^. The analyses were run over 125 interactions to obtain the individual scaled Elo-rank (see Table 2), which was highly stable (0.992) over the study period.

**Figure 1:**
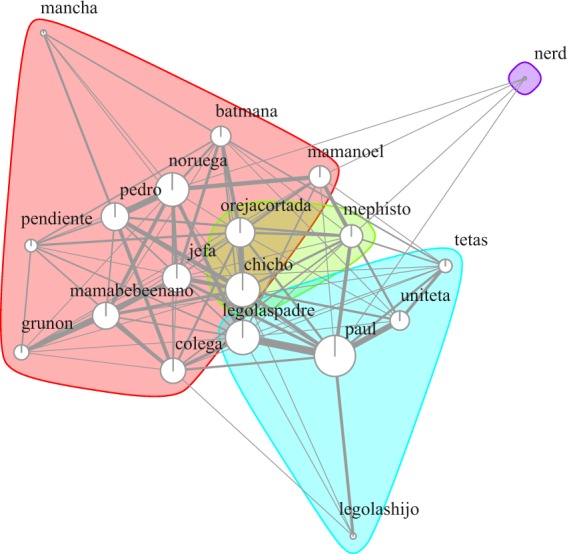
Social network of the group of wild Barbary macaques located in Gibraltar and participating in this study. Weighted edges and nodes are proportional to the individual strength in the network (i.e. the sum of all edge weights connected to the node). Communities are shown with different colours (see Farine & Whitehead^89^). Social network analyses (and the resulting Fig. 1) were run in R, using the vegan (version 2.5-3), asnipe (version 1.1.10) and igraph (version 1.2.1) packages (Oksanen *et al*.^86^; Farine^87^; Csardi & Nepusz^88^).

We further determined the social network based on hourly scans of the group, in which we recorded the spatially closest partner of each individual. We then used these measures to build an undirected weighted matrix and run social network analyses, using the following packages in R: vegan (version 2.5-3)^86^, asnipe (version 1.1.10)^87^, and igraph (version 1.2.1)^88^. Social network analyses assessed individuals’ Eigenvector centrality (i.e. the sum of the centralities of an individual’s neighbours, a measure of the importance of each individual “as a social hub”) and strength (i.e. the sum of all edge weights connected to the node, which represents the expected total interaction rate for each individual^50,89^; see Table 2; Fig. 1). As Eigenvector centrality and strength were correlated, only centrality was used in the analyses (see below).

During the first weeks of observations, the Experimenter (A.L.C.) also assessed monkeys’ reaction to a novel human, by adapting the human orientation test described by Damerius and colleagues^64^. In particular, the Experimenter estimated the individual reaction to a novel human by attributing each individual a value from 1 to 5, based on the monkey’s reaction to the Experimenter, when he approached them in the first days of the habituation (1 being a very positive reaction, e.g. maintaining proximity and lip-smacking to the Experimenter; 3 being a neutral reaction, e.g. resting or moving calmly; and 5 being a very negative reaction, e.g. quickly fleeing and showing aggressive behaviours toward the Experimenter).

**Figure 2:**
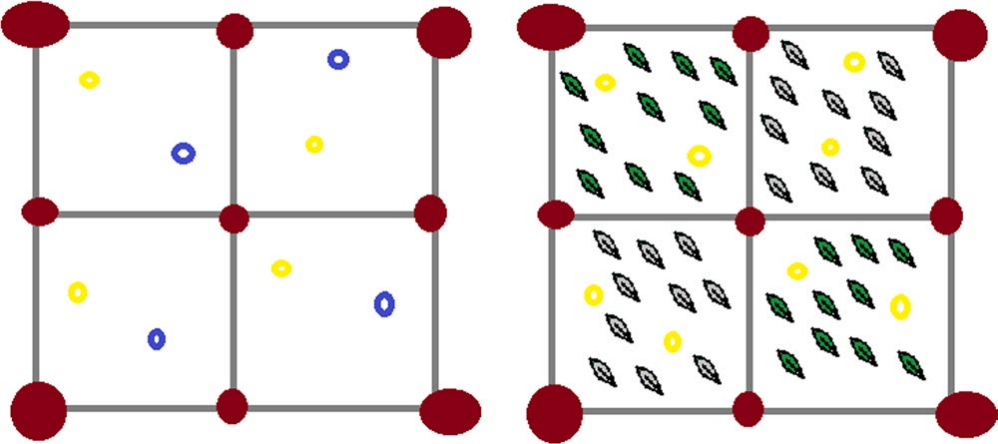
Set-up of the neophobia task administered to the wild group of Barbary macaques tested in Gibraltar. The full ovals represent stones. On the left, set-up for the first and second conditions of the neophobia task. The light/yellow empty ovals represent banana slices, and the dark/blue empty circles represent blue- (in the first condition) or red-dyed (in the second condition) banana slices. On the right, set-up for the third and fourth conditions of the neophobia task. The dark/green rhombs represent local leaves, and the light/silver rhombs represent silver (in the third condition) or yellow (in the fourth condition) pieces of salt-dough.

We further assessed neophobia levels with a specific task. We prepared a testing arena of 4 ×4 meters, dividing it into 4 identical squares marked with stones (see Fig. 2). Inside this arena, we distributed 8 slices of banana. In the first condition, we distributed 2 banana slices per square, half of them having been previously dyed with blue colour. By monitoring the reaction to novel coloured food (as compared to familiar non-coloured food), we could assess individual neophobic levels. In particular, we operationalized neophobia as the number of familiar food items eaten, out of the total number of food items eaten by the individual (i.e. a value closer to 1 indicate more neophobia). The second condition was identical to the first one, but food was dyed with a different colour (i.e., red). In the third condition, we distributed 2 banana slices per square, with two non-adjacent squares having been first covered with local leaves, and the 2 other squares having been covered with silver pieces of salt-dough, looking like leaves. We monitored the reaction to novel objects (as compared to familiar leaves), by operationalizing neophobia as the number of food items eaten from the squares with leaves, out of the total number of food items eaten by the individual. The fourth condition was identical to the third one, but pieces of salt-dough were painted yellow. We ran 20 sessions for each of the four conditions. As soon as the banana slices had been positioned in the arena, we video-recorded and later coded all the individuals entering the testing arena and retrieving food, specifying the colour of the food retrieved (i.e. dyed or not), or the position (i.e. square with leaves or salt-dough). Individual levels of neophobia are summarized in Table 2. Please note that food colour had no odour and no taste.

**Figure 3:**
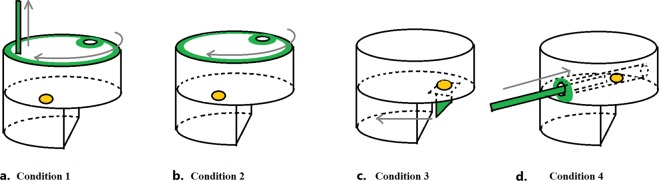
Set-up of the innovation task administered to the wild group of Barbary macaques in Gibraltar, for (**a**) Condition 1, (**b**) Condition 2, (**c**) Condition 3 and (**d**) Condition 4. The light grey/yellow full oval represents food. Dark grey/green parts represent the functional parts of the boxes. Grey arrows represent the movements needed to access the food in the four conditions.

The innovation task was administered after the neophobia task and consisted of four different conditions (see Fig. 3). In each condition, up to three identical boxes (approximately 40 ×40 ×40 cm) were tied to poles, approximately 1.5 m from each other, for several consecutive days (up to two hours a day). The presence and position of several identical boxes prevented complete monopolization by higher-ranking individuals. All boxes were baited with a highly liked food reward (i.e. a peanut). We considered a trial to start when one individual (i.e. the subject) came within 1 m of a box, except if (i) the box had already been emptied by another individual and not yet refilled, or (ii) the individual approached the box from the back (so that it was impossible to interact with the box in any way). A trial was considered to end if the individual retrieved the food or left the box (i.e. went further than 1 m from the box). No effort was made to control which individuals approached the boxes and participated in the tasks^40^. In all conditions, the foraging box was completely transparent (so that food was visible), with the exception of the green functional parts (i.e. the peg, lid, tab or stick, depending on the condition; see Thornton & Samson, 2012, for a similar apparatus).

In the first condition, food could be accessed through a 2-step procedure, by first removing a peg, and then rotating the green upper lid. In this way, the upper hole aligned to the food inside the box, and the subject could retrieve the food (Fig. 3a). After 41 trials, however, none of the individuals could successfully remove the peg. Therefore, all the following conditions were run without the peg, and food could be accessed with a 1-step procedure. In the second condition, subjects had to rotate the green upper lid, as above, to retrieve the food (Fig. 3b). In the third condition, food could be accessed by pulling a little tab, so that food would fall out of the box (Fig. 3c). In the fourth condition, food could be accessed by pushing it out of a green cylinder with a stick (Fig. 3d). To facilitate the solution of the task, the stick had been partially inserted in the tube. All actions required to solve the task (i.e. sliding, pulling and pushing) belong to the natural behavioural repertoire of the species, and prior habitual tool use was not a prerequisite to solve these tasks^40,75^. For each condition, we ran a total of 41, 94, 125 and 136 trials, respectively. As trials were opportunistically run by placing the boxes in the group, the number of trials differed across conditions.

All experimental protocols were discussed and approved by the Helping Hand Trust in Gibraltar, which allowed us access to the macaques. The procedures were also approved by the ethics committees of La Montagne des Singes (Kintzheim, France), the Nürnberg Zoo (Germany) and the Kyoto University (Japan). All methods were carried out in accordance with the national regulations of the country in which the study took place.

### Coding and statistical analyses

All trials were video-recorded with two video-cameras, one providing an overview of all the boxes, and the other one a close-up image of each foraging box. By watching all the videos, we later coded the identity of each subject participating in a trial, trial duration, whether the subject retrieved the food, time spent manipulating functional and non-functional parts of the box (as a measure of exploration), and proportion of time spent manipulating only functional parts of the box (to assess whether manipulations mainly implied functional actions, or rather a mix of functional and non-functional actions as in trial-and-error strategies). We further coded the number of attempts made by the subject to try and directly reach for food through the transparent plexiglas (as a measure of inhibition), and latency to touch the box for the first time (measured from the beginning of the trial, i.e. from the moment the subject came within 1 m of a box, until the subject touched the box). If the subject never approached the box during the trial, latency was considered as the total duration of the trial (i.e., from the moment the subject came within 1 m of the box, until the subject left). No latency measures were taken for subjects who did not come within 1 m of a box. Finally, we noted whether other individuals were present (i.e. within 1 m from the boxes) to test whether the presence of conspecifics facilitated the solution of the task. A second observer blind to the hypotheses of the study re-coded 20% of all the trials to assess individuals’ success, latency to approach the box, exploration, manipulation of functional parts of the box, inhibition and presence of other individuals at the boxes. Inter-observer reliability was very good (for success, inhibition and presence of others, N = 79, and Cohen’s κ = 1, κ = 0.681 and κ = 0.883, respectively; for latency, exploration and functional manipulation, *p* < 0.001, and *r*_*s*_ = 0.992, *r*_*s*_ = 0.998 and *r*_*s*_ = 0.923, respectively).

Analyses were conducted using multilevel-ordered logit models, always including a varying intercept by subject identity to correct for repeated observations. A first set of models assessed the factors affecting variation in the ability to obtain food from the novel foraging boxes. We compared a null intercept-only model (M1.0) to models obtained by adding individual characteristics (i.e. sex, age and rank: M1.1; or centrality, neophobia and reaction to humans: M1.2), measures of the individual behaviour during the task (i.e. latency to approach the box: M1.3; exploration of the box: M1.4; manipulation of the functional parts of the box: M1.5; inhibition: M1.6; presence of others at the boxes: M1.7), or experimental contingencies (i.e. experimental condition and number of trials in which the individual participated: M1.8) as fixed effects (see Table 1). In all models, fixed effects were not correlated.

Given the relatively small sample size, statistical analyses were run with a Bayesian approach, using the rethinking package^90^ in R (version 3.2.3). In all models, we used weakly informative priors and estimated parameters with RStan^91^, running 3 Hamiltonian Monte Carlo chains in parallel (with 10000 samples, half of which were warm-up samples). Convergence was suggested by a high effective number of samples (always ≥1878) and Rhat estimates of 1.00^90^. We then selected models based on the lowest Widely Applicable Information Criteria (WAIC, a measure of sample deviance), and further calculated Akaike weights to estimate the relative probability that different models will best predict future data.

If the selected best models included measures of individual behaviour (i.e. latency to approach the box, exploration of the box, manipulation of its functional parts, inhibition or presence of others near the boxes), a further set of models was run to assess whether the behaviour shown in the task was in turn affected by the individual characteristics of the subject (i.e. sex, age and rank: M2.1/M3.1; or centrality, neophobia and reaction to humans: M2.2/3.2). We then compared this second set of models to a null intercept-only model (M2.0/M3.0; see Table 1). Finally, we assessed whether individual performance was consistent across conditions (i.e. whether subjects who were more successful in one condition were also more successful in the other conditions). We therefore ran Spearman correlations between the individual proportion of successful trials in conditions 2-3, 2–4 and 3–4.

## Results

**Figure 4:**
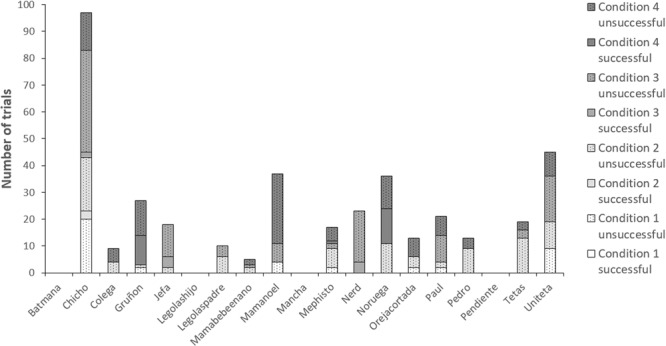
For each individual in the group of wild Barbary macaques tested in Gibraltar, number of trials in which the subject participated and obtained the food, in each of the four conditions.

Out of the 19 individuals in the group, 15 participated in at least one of the conditions. Of these 15 individuals, only 6 individuals solved the task at least once (see Table 2; Fig. 4). The proportion of successful trials by each individual did not correlate across conditions (Conditions 2–3: N = 19, *r*_*s*_, = 0.474, *p* = 0.164; Conditions 2–4: N = 19, *r*_*s*_, = −0.102, *p* = 1.000; Conditions 3–4: N = 19, *r*_*s*_, = −0.186, *p* = 0.874).

**Figure 5:**
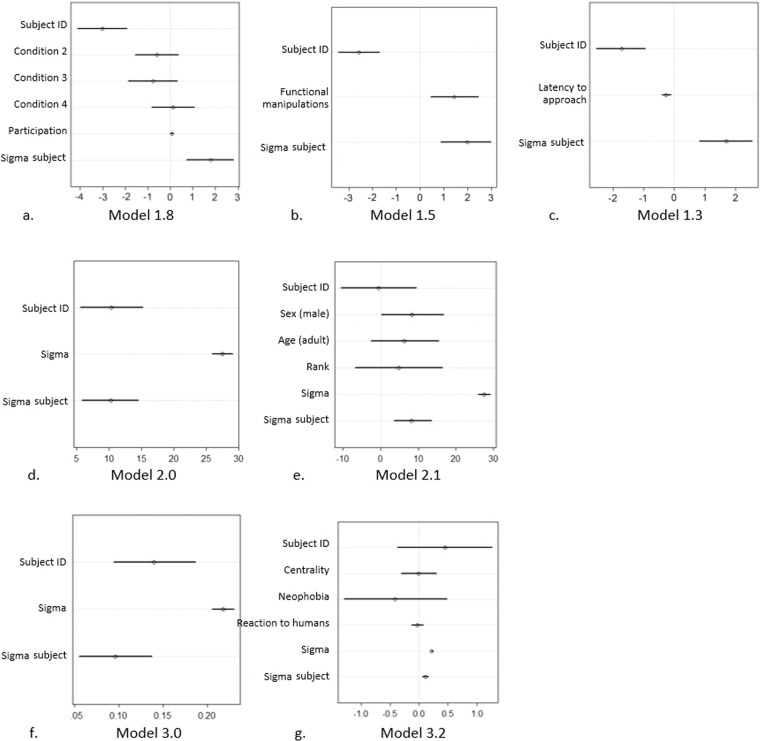
For each set of models, estimates of the best models: (**a**) M1.8, (**b**) M1.5 and (**c**) M1.3; (**d**) M2.0 and (**e**) M2.1; and (**f**) M3.0 and (**g**) M3.2 (see Table 3 for more details).

In set 1, we compared different models to assess which factors affect individuals’ ability to obtain food from the novel foraging boxes. M1.8, M1.5 and M1.3 had the lowest WAIC and the highest model weight (0.47, 0.26 and 0.22, respectively), showing that experimental contingencies (M1.8), manipulation of the functional parts of the box (M1.5) and latency to approach the box (M1.3) all contributed to the model fit (see Table 3; Fig. 5a–c), while the other fixed effects only had a very marginal effect (i.e. exploration; sex, age, rank; centrality, neophobia and reaction to humans) or no effect at all (i.e. inhibition; presence of others at the boxes). In particular, performance varied across conditions, with probability of success being lower in Condition 1 than in all the other conditions. Participating in a higher number of trials also slightly but positively affected performance (M1.8: *β* = 0.07, 89% Prediction Interval [PI] = 0.02 to 0.12). Moreover, better performance in the innovation task was predicted by a higher proportion of functional manipulations (M1.5: *β* = 1.43, 89% PI = 0.44 to 2.45), and by a lower latency during the task (M1.3: *β* = −0.26, 89% PI = −0.40 to −0.10).

**Table 3:** Sets of models, ordered with the smallest WAIC (Widely Applicable Information Criteria) first.

Set	Model	Fixed effects included	WAIC	pWAIC	dWAIC	weight	SE	dSE
1	**M1.8**	**Condition, number of trials**	197.6	8.2	0.0	0.47	21.68	NA
	**M1.5**	**Functional manipulation**	198.8	7.6	1.2	0.26	19.68	6.78
	**M1.3**	**Latency to approach**	199.2	8.9	1.5	0.22	22.35	9.65
	M1.4	Exploration	204.7	7.9	7.0	0.01	21.85	4.93
	M1.2	Centrality, neophobia, RH	205.3	7.6	7.7	0.01	22.05	4.54
	M1.1	Sex, age, rank	205.6	7.6	8.0	0.01	21.81	4.63
	M1.0	—	206.6	7.2	8.9	0.01	21.46	4.49
	M1.7	Presence of others at the box	208.1	8.0	10.4	0.00	21.86	4.72
	M1.6	Inhibition	208.7	8.1	11.0	0.00	22.01	4.20
2	**M2.0**	—	3781.6	79.8	0.0	0.52	320.10	NA
	**M2.1**	**Sex, age, rank**	3781.9	78.6	0.4	0.43	318.47	2.28
	M2.2	Centrality, neophobia, RH	3786.3	80.4	4.7	0.05	323.80	3.80
3	**M3.0**	—	−69.2	12.9	0.0	0.52	38.69	NA
	**M3.2**	**Centrality, neophobia, RH**	−68.5	13.9	0.7	0.36	38.54	1.35
	M3.1	Sex, age, rank	−66.3	14.3	2.9	0.12	38.32	2.05

Each set of models has the same dependent variable (i.e., success in the innovation task in set 1, latency to approach the box in set 2, and proportion of functional manipulations in set 3). The best models in each set are presented in bold. For each model, we present the fixed effects included (apart from the intercept and an intercept by subject identity, which were included in all models), WAIC, estimated effective number of parameters, relative difference with the WAIC for the top-ranked model, Akaike weight, standard error for the WAIC computations and for the difference with the WAIC for the top-ranked model. RH stands for reaction to humans.

We therefore ran two further sets of models, with latency to approach and functional manipulation of the box as dependent variables in set 2 and 3, respectively. In set 2, the WAIC was highest in M2.3, and slightly lower in M2.0 than in M2.1 (see Table 3; Fig. 5d,e). As the model weight was high in both M2.0 and M2.1 (0.52 and 0.43, respectively), we examined the estimates of both models. In M2.1, sex appeared to play the main effect on latency, with males having a higher latency than females (*β* = 8.37, 89% PI = 0.15 to 16.71). In set 3, the WAIC was highest in M3.1, and lower in M3.0 than M3.2 (see Table 3; Fig. 5f,g). As both M3.0 and M3.2 had a high weight (0.52 and 0.36, respectively), however, both models were examined. The model estimates in M3.2, however, suggested that centrality and reaction to humans had no clear effect on the proportion of time spent in functional manipulations of the boxes.

## Discussion

In this study, we investigated inter-individual differences in innovation in a wild population of Barbary macaques. Although most individuals participated in the task, only few were successful, with no consistency across tasks. In particular, success in the novel task was mainly affected by the experimental contingencies and the behavioural strategies used during the task. Individuals were more successful in the 1-step conditions, if they participated in more trials, showed little latency to approach the boxes and mainly manipulated functional parts of the box. In contrast, individual characteristics of the subjects (i.e. sex, age, rank, centrality, neophobia and reaction to humans), as well as inhibition and presence of others at the boxes had little to no effect on the individuals’ ability to innovate.

In our study, none of the subjects could spontaneously use a 2-step strategy to solve the first condition of this task. To avoid a decrease in motivation and ensure the participation of wild individuals, we refrained from administering more trials in this condition. However, it is possible that some individuals would have mastered this task, if given more time. Another possibility, however, is that 2-step strategies are cognitively very demanding and beyond the ability of our study taxon. Indeed, evidence of spontaneous innovation in multi-step problems is to date limited to very few species like great apes^28,92–94^, parrots and corvids^53,95–100^. In line with this, evidence of innovation was found in all the other conditions in which 1-step strategies were required.

Our study further revealed inter-individual differences in innovation rate, which largely depended on the behavioural strategy used during the task. The probability to innovate was higher when subjects showed shorter latencies to interact with the box (with females having longer latencies) and a higher proportion of functional manipulations (in line with our predictions; see Table 1). These results suggest that manipulating the functional parts of the foraging boxes (rather than manipulating the box in general) was a better predictor of innovation in wild Barbary macaques. One may speculate that macaques did not simply obtain food by blindly exploring the box, but focused on the functional parts of it to solve the task. If this was the case, subjects unlikely relied on colour to detect the functional parts of the boxes, as they did not transfer their knowledge across conditions (i.e., in which different functional parts were coloured in the same way). However, it is also possible that, as functional manipulations were necessary to solve the task, individuals simply explored the boxes randomly, but only when they happened to explore the functional parts, the boxes got open. Unfortunately, our data do not allow us to differentiate between these two explanations.

Overall exploration of the box (i.e. including manipulation of non-functional parts), in contrast, did not predict innovation. In the past, studies on innovation have found a positive relationship between exploration and innovation in captive primates^20^ and other species (e.g. birds^20,53,56,59^, carnivores^38,39^; see^2^). Differences with previous studies, however, can be easily explained by the fact that exploration has been often operationalized in different ways (e.g. number of different exploratory or functional behaviours during the innovation task, number of different objects/tasks manipulated during other tasks, time spent interacting with the box^18,19^). Even more importantly, most previous studies on innovation have not differentiated between functional and non-functional manipulations of the testing boxes^18^.

Interestingly, individuals showed no consistency across tasks, with some monkeys innovating in one condition, but failing to solve the others. This result is interesting for several reasons. Firstly, it confirms that the contingent behavioural strategies used, rather than specific characteristics of the subject (e.g. sex, age, rank, centrality, neophobia, reaction to humans), determine whether the subject will be successful in a specific task. However, it should be noted that more precise measures of reaction to humans (e.g., exposing monkeys to more novel humans, instead of just one, as we did in this study) might have provided different results. Secondly, our study suggests that there are no consistent individual differences in how likely animals are to innovate. Rather, individuals may come up with the correct behavioural strategy in one context, but fail to do the same in other contexts. Therefore, the ability to innovate may be context-dependent, as has been proposed for neophobia^101^. This would also explain why studies on innovation often provide contrasting results on the effect of individual characteristics on innovation^18^. Moreover, these results suggest that innovation may be spread across conspecifics regardless of their individual characteristics. Finally, our results suggest that macaques may have little ability to generalize across contexts. Across conditions, functional parts of the box were always painted with the same colour, but this cue was not used by macaques to perform functional manipulations across conditions.

The probability of showing inhibitory problems (i.e. directly trying to reach for food through the transparent parts of the foraging box) had no effect on innovation rate (see Table 1). In this study, innovation was operationalized as the solution to a novel problem, in that individuals had to interact with a novel foraging box to reach for food. However, innovation can also imply the novel solution to a familiar problem^1,2^, which requires the inhibition of old strategies when they become inefficient^24^. Therefore, it is possible that inhibitory levels predict innovation only in the second kind of tasks, or when the solution of the task requires planning^28^, as inhibitory demands are higher^102^.

Similarly, the probability to innovate was not related to the presence of others. These results suggest that social facilitation played no important role in this task, in contrast to our predictions (see Table 1). Some studies, for instance, have shown that the mere presence of conspecifics might have a positive effect on individuals’ tendency to innovate, even when individuals are not actively learning innovative solutions from others. In particular, the presence of conspecifics may encourage individuals to be less neophobic and more exploratory, both in primates^103,104^ and other species (e.g. birds^73,105^, fish^106^; see^102^). Given that social facilitation is probably highly context-dependent^107,108^, more studies are needed to understand whether the cognitive demands of this task or other factors explained the lack of social facilitation in this study. For instance, it is possible that macaques’ participation in the task (rather than successful performance) might be affected by the presence of others at the boxes. In the future, studies should better disentangle whether and how exactly others affect participation and performance in innovation tasks.

Overall, our results provide no support to existing evolutionary hypotheses on the emergence of innovation, and only partly confirm previous studies on innovation in captive primates and other taxa. In this respect, they importantly contribute to our understanding of innovation, by extending a consolidated experimental approach to the study of wild primates. To provide a more stringent test of current evolutionary hypotheses on the emergence of innovation and better understand the evolutionary origins of human innovation, it will be especially important to conduct more studies on innovation in wild primates. In particular, future studies will need to include a higher number of individuals in different captive conditions, to allow direct comparisons between wild and captive conspecifics, and better understand the role played by captivity on the ability to innovate. Moreover, future studies should include more species tested with the same procedures, to directly compare how the socio-ecological characteristics of a species affect innovation. Particularly interesting would be the comparison of different macaque species, to understand whether rank or centrality play different roles depending on the species dominance styles^109,110^. Furthermore, inter- and intra-specific comparisons on innovation should ideally use multiple foraging tasks, to further assess consistency across tasks, and better understand the role played by inhibition on the ability to innovate. Integrating the study of social behaviour to the investigation of innovation will surely provide novel insights into this fascinating but still overlooked topic.

## Supplementary information

Supplementary information.

## Data Availability

All data are available as supplementary material.
